# Determinants of clozapine and olanzapine dose-to-concentration ratio: impact of age, sex, BMI, smoking and inflammation

**DOI:** 10.1192/j.eurpsy.2026.11564

**Published:** 2026-07-31

**Authors:** P. Slana, I. Taskova, N. Safarova, D. Fialova

**Affiliations:** 1 Department of Pharmacology and Toxicology, Masaryk University, Faculty of Pharmacy, Brno; 2 Department of Clinical Pharmacy, Psychiatric Hospital Bohnice, Prague; 3 Department of Social and Clinical Pharmacy, Charles University, Faculty of Pharmacy, Hradec Králové; 4 Third Faculty of Medicine, Charles University, Prague; 5 Research Center for First-episode SMI, National Institute of Menthal Health, Klecany; 6 Department of Social and Clinical Pharmacy, Charles University, Faculty of Pharmacy; 7 Department of Internal Medicine and Geriatrics, Charles University, First Faculty of Medicine, Prague, Czech Republic

## Abstract

**Introduction:**

Therapeutic drug monitoring (TDM) is a valuable tool for capturing interindividual variability in antipsychotic pharmacokinetics and optimising pharmacotherapy in patients with mental illnesses. Although clinical response remains the primary determinant of effectiveness, plasma concentrations—varying up to 45-fold (Rajkumar et al. Int Clin Psychopharmacol 2013)—are stronger predictors of response than dose alone. This variability is influenced by age, sex, genetics, comorbidities, and environmental factors such as smoking (Hiemke et al. Pharmacopsychiatry 2018).

The dose-to-concentration ratio (D/C) reflects drug clearance and reduces the confounding effect of dose variability. Despite the availability of effective antipsychotics, up to 60% of patients with schizophrenia remain treatment-resistant or experience poor antipsychotic tolerability (Howes et al., Mol Psychiatry 2022; Huhn et al., Lancet 2019). These challenges highlight the unmet need for strategies to improve both efficacy and tolerability.

**Objectives:**

This study aimed to assess the relationship between D/C age, sex, BMI, smoking status, and CRP in patients treated with clozapine or olanzapine, and to compare these determinants.

**Methods:**

The retrospective study was conducted at Psychiatric Hospital Bohnice in Prague, from October 2023 to August 2025. The population included inpatients receiving clozapine (n = 88; 56 men; mean age 42.4 years, range 18–78; 71 with F20.x diagnosis) or olanzapine (n = 83; 49 men; mean age 42.4 years, range 17–84; 30 with F20.x diagnosis). This study was also conducted within the framework of the NETPHARM project, which aims to optimise pharmacotherapy in frail and multimorbid older adults.

Group differences were assessed using the Wilcoxon test. Other analyses were performed using Spearman’s coefficient in R software (v4.4.2).

**Results:**

For clozapine, higher age (r = –0.31, p = 0.003) and CRP (r = –0.18, p = 0.089) were associated with lower D/C, while smoking correlated positively (r = 0.35, p < 0.001). Males had higher D/C than females (0.48 vs. 0.38, p = 0.004). No association with BMI was observed (r = 0.08, p = 0.433). For olanzapine, no associations were found with age, BMI, or CRP, while smoking correlated positively (r = 0.24, p = 0.026), and males had higher D/C than females (0.44 vs. 0.27, p = 0.004).

**Conclusions:**

Clozapine D/C was influenced by age, sex, smoking, and CRP, whereas olanzapine D/C was affected only by sex and smoking. These patterns reflect differences in pharmacokinetic pathways (Hiemke et al. Pharmacopsychiatry 2018) and underline the pivotal role of TDM in psychiatry. In line with the NETPHARM project, the results highlight that older adults with comorbidities may particularly benefit from careful TDM of antipsychotics, as they are at the increased risk of drug accumulation and toxicity.

**Disclosure of Interest:**

P. Slana: None Declared, I. Taskova Grant / Research support from: NETPHARM project (CZ.02.01.01/00/22_008/0004607), co-funded by the European Union., N. Safarova: None Declared, D. Fialova Grant / Research support from: NETPHARM project (CZ.02.01.01/00/22_008/0004607), co-funded by the European Union

